# Use of Antibiotics for Maintenance of Axenic Cultures of *Amphidinium carterae* for the Analysis of Translation

**DOI:** 10.3390/md15080242

**Published:** 2017-08-01

**Authors:** Chieh-Lun Liu, Allen R. Place, Rosemary Jagus

**Affiliations:** Institute of Marine and Environmental Technology, University of Maryland Center for Environmental Science, 701 E. Pratt Street, Baltimore, MD 21202, USA; liuh@umces.edu (C.-L.L.); place@umces.edu (A.R.P.)

**Keywords:** *Amphidinium carterae*, axenic cultures, bacterized cultures, antibiotics, protein synthesis inhibitors

## Abstract

Most dinoflagellates in culture are bacterized, complicating the quantification of protein synthesis, as well as the analysis of its regulation. In bacterized cultures of *Amphidinium carterae* Hulbert, up to 80% of protein synthetic activity appears to be predominantly bacterial based on responses to inhibitors of protein synthesis. To circumvent this, axenic cultures of *A. carterae* were obtained and shown to respond to inhibitors of protein synthesis in a manner characteristic of eukaryotes. However, these responses changed with time in culture correlating with the reappearance of bacteria. Here we show that culture with kanamycin (50 μg/mL), carbenicillin (100 μg/mL), and streptomycin sulfate (50 μg/mL) (KCS), but not 100 units/mL of penicillin and streptomycin (PS), prevents the reappearance of bacteria and allows *A. carterae* protein synthesis to be quantified without the contribution of an associated bacterial community. We demonstrate that *A. carterae* can grow in the absence of a bacterial community. Furthermore, maintenance in KCS does not inhibit the growth of *A. carterae* cultures but slightly extends the growth phase and allows accumulation to somewhat higher saturation densities. We also show that cultures of *A. carterae* maintained in KCS respond to the eukaryotic protein synthesis inhibitors cycloheximide, emetine, and harringtonine. Establishment of these culture conditions will facilitate our ability to use polysome fractionation and ribosome profiling to study mRNA recruitment. Furthermore, this study shows that a simple and fast appraisal of the presence of a bacterial community in *A. carterae* cultures can be made by comparing responses to cycloheximide and chloramphenicol rather than depending on lengthier culture-based assessments.

## 1. Introduction

*Amphidinium* is a widespread dinoflagellate genus, found in temperate and tropical marine waters, in both free-living and endosymbiotic states. *Amphidinium* appears to be a relatively early evolving lineage of dinoflagellates based on phylogenetic studies of transcriptomes and contains the hallmark dinoflagellate plastid with peridinin pigment [1,2,3,4]. *Amphidinium carterae* Hubert represents the most well-studied dinoflagellate species and has begun to emerge as a “model” for dinoflagellates in research into dinoflagellate genetics, photosynthesis, and polyketide/toxin production (reviewed [5]). *A. carterae* Hubert grows well in culture to high cell densities, is available from culture collections, has a relatively small genome of about 5.9 × 10^9^ bp [6]. In addition, *A. carterae* is considered one of the new target species suitable for commercial exploitation on an industrial scale [7] since it shows resistance to mechanical stress in photobioreactors and because several high value metabolites can be extracted from it [8,9]. 

Dinoflagellates exhibit little transcriptional regulation of gene expression [10,11,12,13], but have been widely demonstrated to show changes in expressed proteins [14,15,16,17,18,19]. Post-transcriptional regulation of gene expression in dinoflagellates has focused on translational control and in particular on the regulation of mRNA recruitment [20,21,22,23]. All nuclear mRNA in dinoflagellates is *trans*-spliced with a 22-nucleotide 5′-spliced-leader sequence [24,25]. In *A. carterae*, the spliced leader RNA introduces a m^7^Gppp cap structure at the 5′ end, any one of the four nucleotides at the second position and additional methylations on downstream bases to give novel multi-methylated caps [21]. Furthermore, eight family members of the cap binding translation initiation factor eIF4E, from three novel clades, have the potential to participate in, or regulate, mRNA recruitment in *A. carterae* [21,22].

Polysome fractionation by velocity centrifugation in sucrose gradients is a routine technique for examining the translating pool of ribosomes in a cell and has been used to monitor translational status under various physiological conditions (reviewed [26]). As the ribosome moves along the mRNA molecule during translation elongation, additional ribosomes can initiate translation on the same RNA molecule, forming polysomes. Since ribosome loading on mRNA is determined by the rate of translational initiation and elongation, polysome analysis can be used to assess the global translational level. Polysome fractionation enables direct monitoring of the efficiency of translation, but can also provide a snapshot of the mRNAs being actively recruited and translated. By treating cells with a translation elongation inhibitor, such as cycloheximide or emetine, elongating polyribosomes can be frozen on the mRNA they are translating and analyzed. The polyribosomes are separated from monosomes, as well as the individual ribosomal subunits by sucrose gradient fractionation. Sequencing the mRNAs in each fraction allows direct determination of the differential translation of individual mRNAs on a genome-wide scale. In addition to studying changes in mRNA recruitment, a polyribosome fractionation protocol can be used to isolate and characterize the translation factors and other proteins associated with mRNA on ribosomes and polysomes. The proteins associated with each fraction can be analyzed by Western blotting or by shotgun proteomics with mass spectroscopy and a translated transcriptome. The most widely used stabilizing reagent used to prevent polysomal run-off for such experiments is the eukaryotic protein synthesis inhibitor cycloheximide. Cycloheximide binds the 60S subunit in the translating 80S ribosome, blocking translation elongation by preventing release of deacylated tRNA from the ribosomal E site after translocation [27]. Footprinting experiments have revealed protection of a single cytidine nucleotide (C3993) in the E-site of the 60S ribosomal subunit [28].

An additional ribosome profiling technique has been developed, in which ribosome protected fragments (RPFs) are generated by RNAse I treatment and analyzed by deep-sequencing [29,30]. A translating ribosome encloses a ~30 nt portion of this mRNA template and protects it from nuclease digestion [31]. These ribosome-protected mRNA fragments can be used to map the positions of ribosomes allowing the determination of ribosome position at single nucleotide resolution. For example, ribosome profiling can be used for determination of ribosome density on a given mRNA molecule or the identification of elements that influence translation initiation rates such as alternative initiation sites, initiation at non-AUG codons and regulatory elements such as upstream open reading frames (uORFs). Although ribosome profiling allows direct identification of ribosome position on a given mRNA molecule, the number of ribosomes associated with a given mRNA is only indirectly estimated by normalizing frequencies of reads in ribosome-associated mRNA over those observed in randomly fragmented mRNAs (total mRNA). To define translated reading frames better, the sites of translation initiation can be revealed using the protein synthesis inhibitor harringtonine to immobilize initiating ribosomes. Harringtonine immobilizes ribosomes immediately after translation initiation begins and results in footprint accumulation at all initiation sites [29,30,32]. Polysome fractionation and ribosome profiling are complementary methods that provide information regarding the number of ribosomes associated with mRNA and position of the ribosome on mRNA, respectively.

To begin polysome studies in *A. carterae*, we needed to optimize use of the translational inhibitors, cycloheximide, emetine, and harringtonine, monitoring their effectiveness by ^35^S-methionine incorporation. Our *A. carterae* cultures were barely responsive to these inhibitors. In contrast, ^35^S-methionine incorporation was severely reduced by addition of chloramphenicol, an inhibitor of bacterial protein synthesis. The lab culture had begun as an axenic culture of *A. carterae* Hulbert from Bigelow National Center for Marine Algae and Microbiota (CCMP 1314) maintained in L1 medium under sterile conditions. However, the response to cycloheximide and chloramphenicol suggested that the culture had not remained axenic although unattached bacteria were not apparent by flow cytometry and attached bacteria were not detected by DAPI staining. Strategies to develop axenic cultures of dinoflagellates usually involve a labor-intensive combination of microfiltration to remove bacteria, treatment with various antibiotic or chemical regimes, collection of phototactile cells and the generation of colonies from single cells [33]. Testing requires that axenic cultures be grown in rich medium and incubated in the dark for three to four weeks in liquid media or on plates to allow any contaminants to grow. Dinoflagellates are universally associated with bacteria in the ocean. Although the number and diversity of associated bacterial genera are variable among different dinoflagellate species (reviewed [34]), they resemble an ordered and structured community rather than a random assemblage of species recruited from the marine bacterial metacommunity likely reflecting a physiological relationship [35,36]. The bacterial group most often associated with dinoflagellates is the Alphaproteobacteria, most frequently from the *Roseobacter* clade and its relatives [36,37,38]. Gammaproteobacteria, Betaproteobacteria, and Cytophaga-Flavobacteria-Bacteroides (CFB) are the other bacterial groups reported to be associated frequently with dinoflagellate species. Within the Gammaproteobacteria, *Marinobacter* spp. and *Alteromonas* spp. appear to have an association with dinoflagellates [37,39].

Although photosynthetic, most dinoflagellates are auxotrophs for B-vitamins, requiring different combinations of three B-vitamins: vitamin B12 (cobalamin), vitamin B1 (thiamine), and vitamin B7 (biotin) [40,41,42]. *A. carterae* is listed as requiring all three B-vitamins [40]. In culture, B-vitamins are provided from the L1 medium [43,44], but can also be provided by unattached or episymbiotic bacteria [45]. As examples, the growth of axenic *Lingulodinium polyedron* grown without B-vitamins can be sustained by *Maravita* spp. (from the Roseobacter clade) and *Marinobacter flavimaris*, both B-vitamin producers [45]. Similarly, the growth of axenic *Gymnodinium catenatum* is stimulated by co-culture with *Roseobacter* spp. and *Marinobacter* spp. [46].

**Figure 1:**
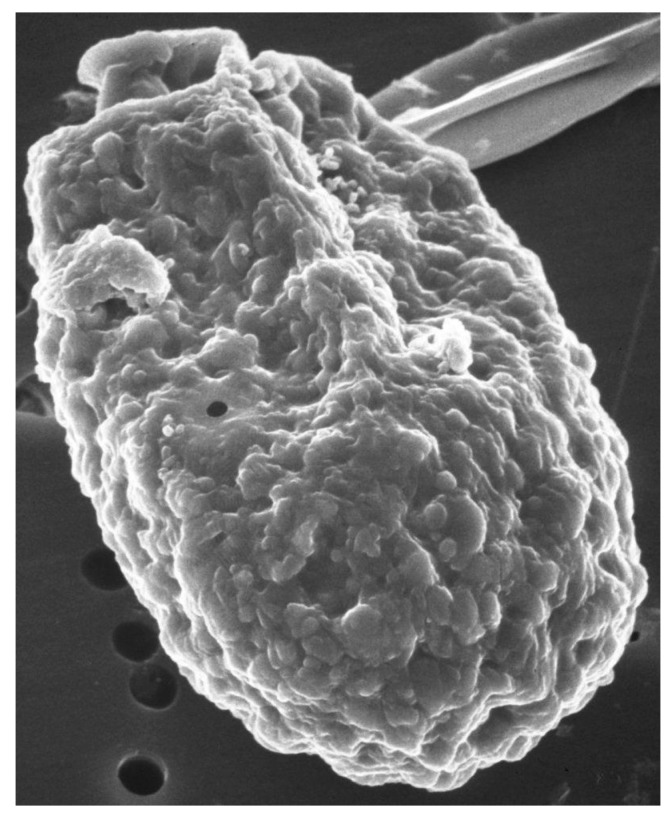
Scanning electron micrograph of *A. carterae.* Scanning electron micrograph of *A. carterae* to illustrate cell surface. From the Smithsonian Collection.

Scanning electron microscope images of *A. carterae*, such as those reported in Aquino-Cruz and Okolodkov [47] and Murray et al. [5] show the cell surface of *A. carterae* to have small, rounded structures of less than 100 nm on the cell surface that provide an extensive surface area (Figure 1). This is covered by an adhesive cell-surface coat, referred to as glycocalyx or “sticky fuzz”, a dynamic and complex surface matrix made up of oligosaccharides covalently bound to proteins and lipids of the plasma membrane [48,49]. Fluorescence and transmission electron microscopy, using various FITC-linked lectins, including concanavalin A (ConA), wheat germ agglutinin (WGA), and soybean agglutinin (SBA), highlighted the complexity of cell-surface glycan assemblages and the potential role as a perfect habitat for bacterial adherents. Low numbers of attached cells might escape detection but eventually multiply sufficiently to affect overall protein synthetic rates.

To avoid the complications of associated bacteria in our investigations of protein synthesis in *A. carterae*, we purchased fresh axenic cultures of *A. carterae* Hulbert from Bigelow National Center for Marine Algae and Microbiota (CCMP 1314) to allow us to optimize the inhibition of translation by cycloheximide and other inhibitors of eukaryotic translation, as well as to enable us to quantitate protein synthetic activity in *A. carterae* and analyze mRNA recruitment.

## 2. Results

### 2.1. Bacterized, But Not Axenic A. carterae Show Atypical Responses to Protein Synthesis Inhibitors

**Figure 2:**
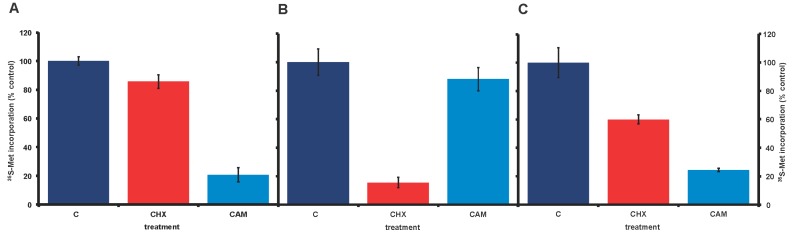
Effect of cycloheximide and chloramphenicol on ^35^S-methionine incorporation in nonaxenic and axenic *A. carterae. A. carterae* cultures were pre-incubated with or without cycloheximide (100 μg/mL) or chloramphenicol (50 µg/mL) for 1 h at 20 °C prior to incubation with [^35^S] methionine (50 μCi·mL^−1^) at 20 °C for 1 h. (A): nonaxenic *A. carterae*; (B): *A. carterae* (CCMP 1314, Bigelow National Center for Marine Algae and Microbiota); and (C): axenic *A. carterae* after maintenance in culture without antibiotics for four months. Without protein synthesis inhibitors (C) (dark blue); plus cycloheximide (CHX) (red); plus chloramphenicol (CAM) (light blue). The incorporation of [^35^S] methionine into TCA precipitable radioactivity was corrected for decay of ^35^S and calculated as cpm per 5 × 10^5^ cells to allow comparison between different experiments. However, incorporation of [^35^S] methionine into TCA precipitable radioactivity is expressed here as a percentage of that in the control culture without antibiotics. Data are presented as the mean ± S.D (*n* = 3 replicates).

In our long-term lab cultures of *A. carterae* (CCMP 1314), incorporation of ^35^S-methionine into trichloracetic acid precipitable material, our measure of protein synthesis, showed little response to the eukaryotic-specific inhibitor of protein synthesis, cycloheximide, at 100 μg/mL (Figure 2, panel A). It seemed possible that this could reflect an unusual characteristic of dinoflagellate ribosomes. However, the bacterial (and plastid) specific protein synthesis inhibitor, chloramphenicol, reduced ^35^S-methionine incorporation to 20% of control values. Although chloramphenicol has the potential to inhibit chloroplast protein synthesis, it seemed unlikely that this would account for 80% of measured *A. carterae* protein synthesis. A likelier explanation was that a significant proportion of the protein synthetic activity observed in our lab *A. carterae* cultures reflected the activity of an associated bacterial community. This was surprising since contaminating bacteria could not be observed. To validate such a conclusion, we obtained fresh axenic *A. carterae* Hulbert (CCMP 1314, Bigelow National Center for Marine Algae and Microbiota, East Boothbay, Maine) and looked again at the effects of the two protein synthesis inhibitors. The incorporation of [^35^S] methionine into TCA precipitable radioactivity was corrected for the decay of ^35^S and calculated as cpm per 5 × 10^5^ cells. The data is expressed as a percentage of the control value. The stocks of protein synthesis inhibitors are dissolved in water at 1000× the final concentration needed, so control cultures have an equivalent volume of water added. In the axenic culture, cycloheximide reduced ^35^S-methionine incorporation to less than 15% of control values (Figure 2, panel B). In contrast, chloramphenicol gave at most a 10% reduction in ^35^S-methionine incorporation. Overall these responses supported our conclusion that our long-term *A. carterae* cultures had become bacterized.

**Figure 3:**
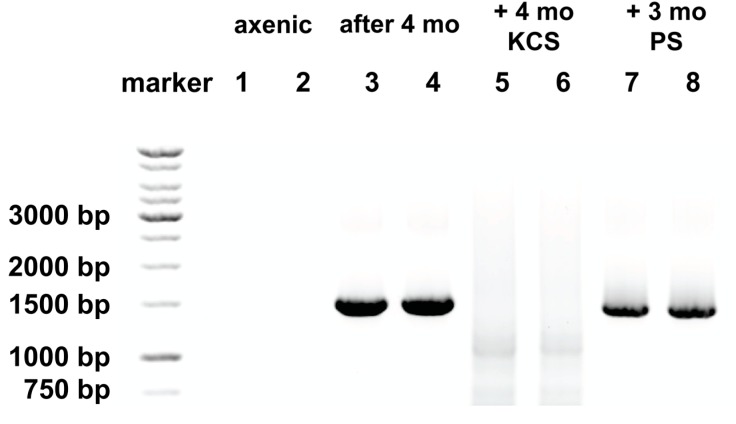
Bacterial rDNA reappears in axenic *A. carterae* cultures over time. The presence of bacterial 16S ribosomal gene (rDNA) was determined in axenic *A. carterae* maintained in culture with and without antibiotics. DNA was isolated from axenic cultures of *A. carterae* Hulbert (CCMP 1314, Bigelow National Center for Marine Algae and Microbiota) and tested for bacterial contamination by amplication of DNA using two universal primers to bacterial 16S ribosomal RNA gene; 27F (5′-AGAGTTTGATCMTGGCTCAG-3′) and Univ1492R (5′-GGTTACCTTGTTAC GACTT-3′) [50,51]. Amplicons were fractionated by 1% agarose gel electrophoresis, visualized by ethidium bromide staining. Images were generated by ChemiDocTMTouch^TM^ (Bio-Rad, Hercules, CA, USA). Lanes 1 and 2: axenic culture, Lanes 3 and 4: the same culture after 4 months in culture without antibiotics; Lanes 5 and 6: the same culture after four months without antibiotics, followed by four months with KCS; Lanes 7 and 8: the same culture after four months in culture without antibiotics followed by three months in PS.

To determine the validity of our inference that the difference in behavior of the axenic culture reflected the absence of a bacterial community, we looked for the presence of bacteria by amplification of 16S rDNA by PCR amplification using two universal primers; the bacterial 16S rDNA primer; 27F (5′-AGAGTTTGATCMTGGCTCAG-3′), and Univ1492R (5′-GGTTACC TTGTTACGACTT-3′), the numbering based on where these fall in *Escherichia coli* 16S rDNA [50,51]. Use of these primers generates amplicons of ~1.5 kbp. By this method, bacteria were not detected in the axenic culture (Figure 3, lanes 1 and 2), but were detected after maintenance of this culture without antibiotics for 4 months (Figure 3, lanes 3 and 4).

Although this universal 16 S primer set is known to amplify mitochondrial and plant chloroplast rRNA genes, the divergent sequences and fragmented nature of the mitochondrial and plastid 16S rRNA genes in *A. carterae* are not amplified by these primers [52,53].

### 2.2. Atypical Responses to Protein Synthesis Inhibitors and Bacteria Reappear with Time in Culture

Later experiments, using the axenic culture after several weeks, showed that the responses to cycloheximide and chloramphenicol had reverted with time, such that higher incorporation of ^35^S-methionine was observed in the presence of cycloheximide, while chloramphenicol gave a pronounced inhibition (Figure 2, panel C). These changed characteristics were reflected in the re-appearance of bacterial rDNA (Figure 3, lanes 3 and 4). 

### 2.3. KCS, But Not PS, Prevents Re-Emergence of Bacteria

There are many reports of the use of antibiotics to generate axenic cultures of dinoflagellates. This may not be possible for heterotrophic dinoflagellates unless they are grown in a rich medium that replaces the nutrients their bacterial associates can provide. However, for photoautotrophic dinoflagellates, it is less of a challenge. A laboratory culture of *A. carterae* that had *Moraxella* and *Pseudomonas* Group IV associated with it was grown in gentamicin, chloramphenicol, and ciprofloxacin [54]. This antibiotic mix failed to totally eliminate these bacteria. Although the bacteria could not be detected 24 h after addition of the antibiotics, after a week, the same bacteria could be found again. Even if successful at eliminating associated bacteria, the continual presence of antibiotics might negatively affect growth and/or otherwise impact the physiological properties of the algae. Soffer et al. [55] compared 13 different antibiotic formulations on growth and presence of bacterial contaminants in liquid cultures of *Symbiodinium*. Certain antibiotics such as erythromycin, doxycycline, tetracyclin and chloramphenicol negatively impacted *Symbiodinium* growth. Other antibiotics, such as kanamycin, ampicillin, streptomycin, and G418 did not inhibit *Symbiodinium* growth, but individually failed to prevent bacterial contamination. A combination of penicillin and streptomycin reduced *Symbiodinium* growth somewhat, but could not eliminate bacterial growth. A combination of kanamycin (50 μg/mL), ampicillin (100 μg/mL), and streptomycin (50 μg/mL) eliminated bacterial contamination without impacting *Symbiodinium* growth. Similarly, Hao et al. were able to maintain axenic cultures of *A. carterae* with a combination of kanamycin, neomycin, and streptomycin and found that this not only prolonged the duration of exponential growth, but also increased the maximum density achieved by 25% [40].

The study by Soffer et al. using antibiotics to eliminate bacterial contamination from a dinoflagellate culture was the most comprehensive we had seen [55]. The kanamycin/ampicillin/streptomycin mix identified as the most effective by Soffer et al. has been used by others to successfully develop axenic *Symbiodinium* strains for introduction into a cnidarian host, *Aiptasia*, as a symbiont [56]. We adopted this mix, but replaced ampicillin with carbenicillin, a carboxypenicillin. Compared to ampicillin, carboxypenicillins are much more resistant to degradation by β-lactamase enzymes [57]. We compared this mix to the use of 100 units/mL each of penicillin and streptomycin sulfate, antibiotics that are routinely used in the generation of axenic dinoflagellates [33]. Maintenance in an antibiotic mix of kanamycin (50 μg/mL), carbenicillin (100 μg/mL), and streptomycin sulfate (50 μg/mL) (KCS) prevented the reappearance of bacterial rDNA in our cultures even after four months (Figure 3, lanes 5 and 6). However, penicillin and streptomycin (PS) both at 100 μg/mL, did not, with clearly apparent bacterial rDNA signatures (Figure 3, lanes 7 and 8). A faint band can be seen in DNA from cultures maintained in KCS at ~1100 bp and a more diffuse smear at ~700 bp (Figure 3, lanes 5 and 6). This reflects mispriming of the bacterial primer 27F when bacterial rDNA is not present, and under the amplification conditions used. To ensure amplification of all bacterial rRNA, a low stringency annealing temperature of 46 °C was used with high cycle number (40 cycles). This has the potential to generate low levels of misprimed amplicons when authentic targets are not available. It has previously been reported that use of this primer set at low stringency can give rise to cross-kingdom amplification of rRNA genes, as is the case for the Pacific scleractinian coral *Pocillopora damicornis* [58]. Upon inspection of the rRNA gene sequence from *A. carterae*, accession #AF274251.1, areas of (poor) sequence homology with the bacterial primer 27F at 172–190, 655–673, 718–737, and the universal primer 1492R. At low stringency, such as that used here, and without authentic targets to compete, the primer combinations could produce amplicons from *A. carterae* rDNA of approximately 1584 bp (which would not be distinguished from the bacterial rDNA amplicons), 1101 bp, as well as a smeary band between 707 and 717 bp. A faint band at ~1100 bp and smeary band of ~700 bp can be seen Figure 3, lanes 5 and 6 corresponding to this.

### 2.4. KCS, But Not PS, Prevents Atypical Responses to Protein Synthesis Inhibitors, But Does Not Impact the Growth Rate of A. carterae

**Figure 4:**
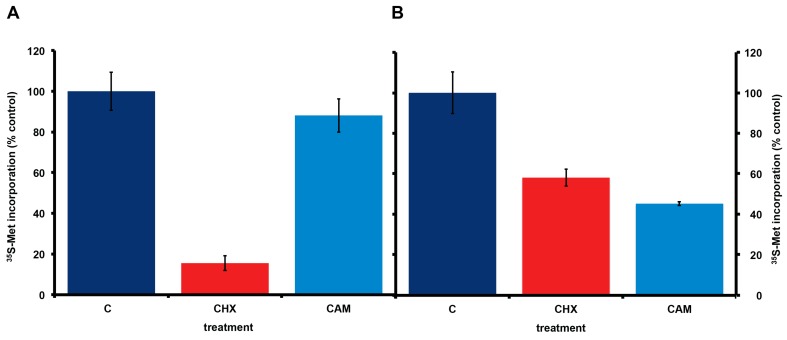
Effect of cycloheximide and chloramphenicol on ^35^S-methionine incorporation in *A. carterae* axenic cultures maintained in different antibiotics. *A. carterae* were pre-incubated with or without cycloheximide (100 μg/mL) or chloramphenicol (50 μg/mL) for 1 h at 20 °C prior to incubation with [^35^S] methionine (50 μCi·mL^−1^) at 20 °C for 1 h. (**A**): cells maintained in KCS for 4 months; (**B**): cells maintained in PS for 3 months. Incorporation of [^35^S] methionine into TCA precipitable radioactivity is expressed as a percentage of that in control cultures without protein synthesis inhibitors. The incorporation of [^35^S] methionine into TCA precipitable radioactivity was corrected for decay of ^35^S and calculated as cpm per 5 × 10^5^ cells. Data are presented as the mean ± S.D (*n* = 3 replicates). Control (dark blue); plus cycloheximide (CHX) (red); plus chloramphenicol (CAM) (light blue).

Consistent with the presence or absence of bacteria, incorporation of ^35^S-methionine in *A. carterae* maintained in KCS shows the expected eukaryotic responses to cycloheximide and chloramphenicol. Cycloheximide reduced ^35^S-methionine incorporation to less than 15% of control values, whereas chloramphenicol gave approximately a 10% reduction in ^35^S-methionine incorporation (Figure 4, panel A). In contrast, in *A. carterae* maintained in PS for three months, cycloheximide only reduced ^35^S-methionine incorporation to ~60% of control, whereas, chloramphenicol reduced it to ~45% suggesting that more than half the protein synthetic activity could be attributed to the bacterial community (Figure 4, panel B). 

**Figure 5:**
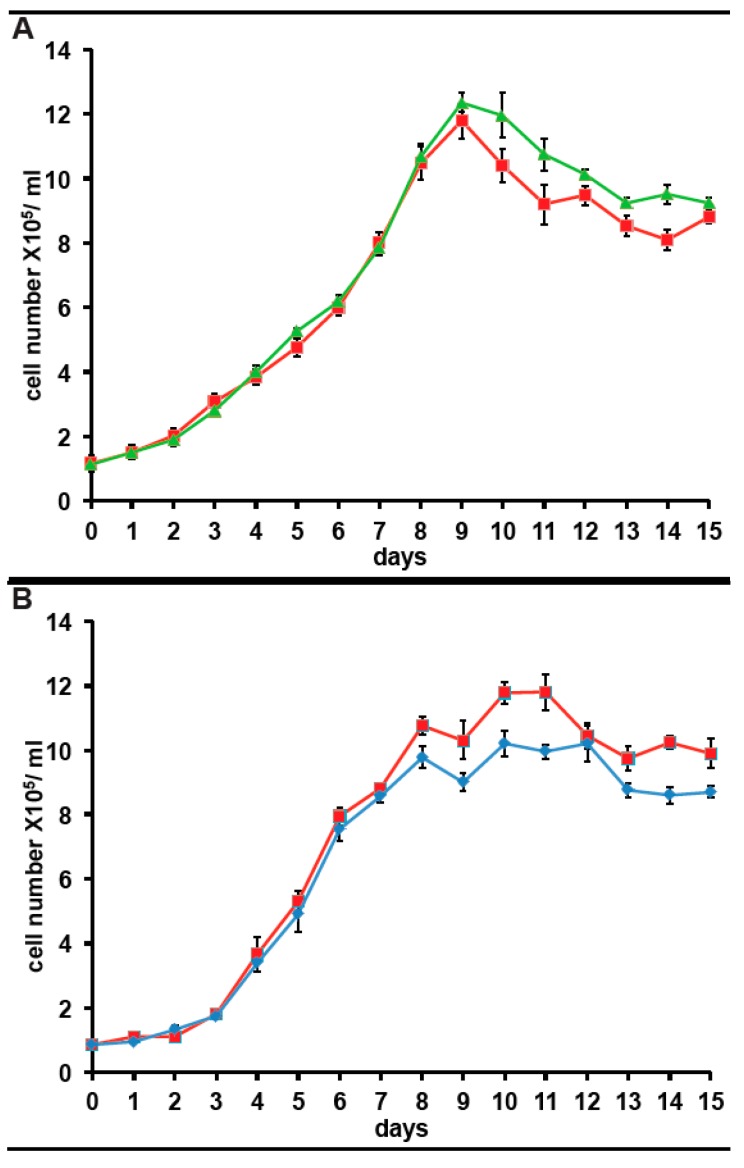
Effect of antibiotics on growth of *A. carterae.* Axenic *A. carterae* maintained without antibiotics for 5 months were exchanged into medium supplemented with or without KCS or PS for 3 days prior to start of growth curve. Growth curves were begun at a starting density of ~1 × 10^5^/mL. Cell counts were measured daily. Data are presented as the mean ± S.D (*n* = 3 replicates). Without antibiotics (red square); plus KCS (green triangles); plus PS (blue diamond). (**A**): comparison of growth with/without KCS; (**B**): comparison of growth with/without PS.

Since we have expressed the incorporation of ^35^S-methionine as a percentage of that in control cultures for clarity, any impact of long term culture in antibiotics on ^35^S-methionine incorporation is not apparent. However, from the data above and other experiments, the ^35^S-methionine incorporation for cells in KCS is 10,081 cpm/5 × 10^5^ cells, adjusted for ^35^S half-life. The corresponding figure is 9867 cpm/5 × 10^5^ cells, adjusted for ^35^S half-life in cells maintained in PS for 3months. Both are 15–20% higher than in cells maintained without antibiotic (8064 cpm/5 × 10^5^ cells, adjusted for ^35^S half-life). Considering that 80% of ^35^S-methionine incorporation in the bacterized cultures reflects the bacterial community, this suggests that protein synthetic activity in *A. carterae* maintained in KCS, is approximately five times higher than in the bacterized cells. It was observed that inclusion of either antibiotic mix gives an initial drop in ^35^S-methionine incorporation presumably as the bacterial count falls (data not shown). However, once the bacterial community is removed, ^35^S-methionine incorporation rises presumably due to the lack of competition from the bacterial community. However, still concerned over whether the antibiotics would impact *A. carterae* growth, we also looked at the effects of the antibiotics on growth. Reverted axenic cultures were exchanged into medium supplemented with or without KCS or PS for three days prior to the start of the growth curves which were begun at a starting density of ~1 × 10^5^/mL. Cultures growing with KCS grew at the same rate as control cultures, but the growth phase persisted longer and achieved higher final densities (Figure 5, panel A). The growth of cultures in PS began to slow down sooner than control cultures and achieved lower final densities (Figure 5, panel A).

### 2.5. Effect of Other Protein Synthesis Inhibitors on ^35^S-Methionine Incorporation in Axenic A. carterae Maintained in KCS

**Figure 6:**
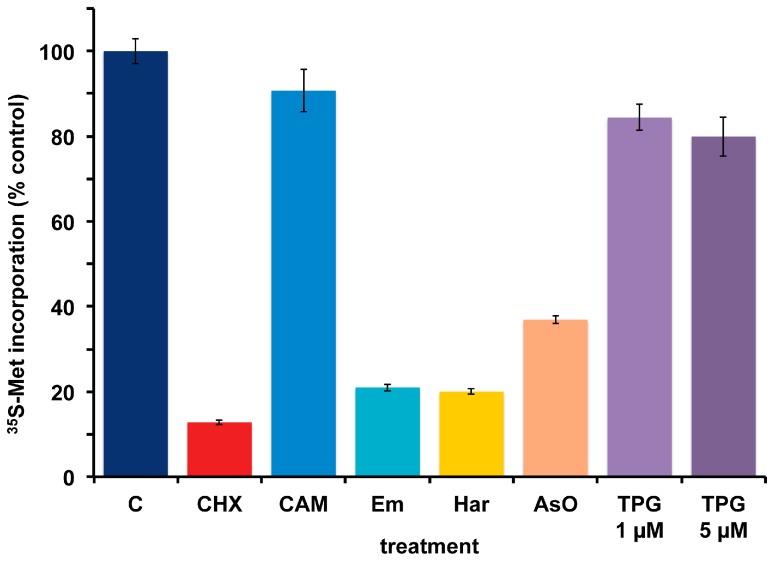
Effect of protein synthesis inhibitors on ^35^S-methionine incorporation in axenic *A. carterae* maintained in KCS. *A. carterae* were pre-incubated for 1 h at 20 °C with or without a range of protein synthesis inhibitors: cycloheximide, 100 μg/mL; chloramphenicol, 50 μg/mL; emetine, 360 μM; harringtonine, 37.6 μM; sodium arsenite, 400 μM; thapsigargin, 1 μM and 5 μM, prior to incubation with [^35^S] methionine (50 μCi·mL^−1^) at 20 °C for 1 h. Incorporation of [^35^S] methionine into TCA precipitable radioactivity is expressed as a percentage of that in control culture without antibiotics. The incorporation of [^35^S] methionine into TCA precipitable radioactivity was corrected for the decay of ^35^S and calculated as cpm per 5 × 10^5^ cells. Data are presented as the mean ± S.D (*n* = 3 replicates). Without antibiotics (dark blue); plus cycloheximide (CHX) (red); plus chloramphenicol (CAM) (light blue); plus emetine (Em) (teal blue); plus harringtonine (Har) (yellow); plus sodium arsenite (AsO) (orange); plus thapsigargin (TPG) (purple).

*A. carterae* maintained in KCS has remained free of bacteria for several months, as measured by the response to cycloheximide and chloramphenicol and lack of PCR-amplifiable 16S rDNA. Over this time, the growth rate, ^35^S-methionine incorporation and final cell densities of the culture has remained consistent and has been used to look at the effects of other agents that inhibit protein synthesis in eukaryotes. This culture has remained consistent in its response to cycloheximide (CHX) which reduces ^35^S-methionine incorporation to less than 15% of control values, as well as to chloramphenicol (CAM) which causes less than a 10% reduction (Figure 6). Other inhibitors of eukaryotic protein synthesis, emetine (Em) and harringtonine (Har) also inhibit ^35^S-methionine incorporation. Cycloheximide, emetine and harringtonine will be used to dissect the regulation of mRNA recruitment in *A. carterae.* Sodium arsenite (AsO), used to mimic oxidative stress, also inhibits ^35^S-methionine incorporation. In contrast, thapsigargin (TPG), a non-competitive inhibitor of the endoplasmic reticulum Ca^2+^ATPase does not. TPG inhibits protein synthesis in metazoans as a consequence of metabolic stress through phosphorylation of the translation initiation factor eIF2 [59].

## 3. Discussion

In order to study translation regulation of gene expression in *A. carterae*, we needed to grow cultures without the associated bacterial community. Strategies to develop axenic cultures of dinoflagellates usually involve a labor-intensive combination of microfiltration to remove bacteria, treatment with chemicals or various antibiotic regimes, collection of phototactile cells and the generation of colonies from single cells [33]. We were able to purchase axenic cultures of *A. carterae* Hulbert from but found that over a few months a bacterial community was re-established. We consider that the most likely source of contamination comes from low levels of bacteria that remain closely associated with the surface of *A. carterae*. Drawing on the work of others, we found that the combination of kanamycin, carbenicillin, and streptomycin, but not the combination of penicillin and streptomycin, allow *A. carterae* to grow without PCR-detectable bacteria over several months. Axenic cultures will allow us to measure protein synthesis rates under different conditions and over the diel cycle, evaluate conditions that stimulate or inhibit protein synthesis and study changes in mRNA recruitment. Other workers in the field looking at translational regulation in dinoflagellates have included prokaryotic inhibitors of protein synthesis like chloramphenicol in the culture medium at the time of their experiment to inhibit incorporation of methionine by the bacterial community present in the cultures [60]. Our data have indicated that this is not an ideal strategy since the dinoflagellates are functioning under sub-optimal nutrient conditions for protein synthesis. It is not known what the long-term effect of antibiotic supplementation are on chloroplast protein synthesis may be. Our KCS-maintained cultures show no signs of deterioration after six months. There is no information in the literature on what fraction of total protein synthesis takes place in the dinoflagellate chloroplast, although it is known that many genes typically located within the chloroplast genome have been transferred to the nuclear genome [61,62] and the rRNA genes are fragmented [52,53]. However, when both cycloheximide and chloramphenicol are included during ^35^S-methionine incorporation, little further reduction is observed over cycloheximide alone, suggesting that chloroplast protein synthesis may be very low.

In addition to providing the axenic cultures we need for our research, this study has demonstrated that a simple and fast appraisal of the presence of a bacterial community in dinoflagellate cultures can be made by comparing responses to the protein synthesis inhibitors cycloheximide and chloramphenicol rather than depending on lengthier culture-based assessments. The labor-intensive generation of axenic dinoflagellate cultures render it important to test for bacterial contamination on a regular basis. This can be done by epifluorescence microscopy or by PCR amplification of 16S rDNA in those dinoflagellate species that have chloroplast 16S rRNA genes amplified by 16S universal primers. A routine and expedient assessment of the response to eukaryotic and prokaryotic inhibitors of protein synthesis could provide a desirable alternative that could be easier for small labs to implement. We plan to follow up these findings by making a comprehensive assessment of the microbiota by combining mass-amplification of bacterial 16S rRNA with universal primers and high-throughput sequencing using MiSeq. Identification of the members of the bacterial community may enable us to choose more effective antibacterial strategies as well as optimize *A. carterae* culture media.

## 4. Materials and Methods

### 4.1. Antibiotics and Protein Synthesis Inhibitors

Chloramphenicol (C0378) and emetine (E2375) were from Sigma (St. Louis, MO, USA). Cycloheximide (94271) was from Amresco (Solon, OH, USA). Sodium arsenite (S-2251) was from Fisher Scientific (Fair lawn, NJ, USA). Thapsigargin (586005) Calbiochem (San Diego, CA, USA). Harringtonine (H0169) was from LKT Labs (St. Paul, MN, USA).

### 4.2. A. carterae Cell Culture

*A. carterae* Hulbert [43] was obtained from the National Center for Marine Algae and Microbiota (CCMP1314, Bigelow National Center for Marine Algae and Microbiota), grown under sterile conditions in filtered L1 medium in 35 ppt seawater containing L1 trace element solution and f/2 vitamin solution [44]. Cultures were grown at 20 °C with 14:10 light: dark cycle with a photon flux density of 35 μmol·m^−2^·s^−1^. Cultures were supplemented with or without kanamycin (50 μg/mL)/carbenicillin (100 μg/mL)/streptomycin (50 μg/mL) (KCS), or 100 units/mL of penicillin and streptomycin sulfate (PS). Cultures were counted daily to monitor growth with cells collected at mid-day. Cell density was maintained at 4–6 × 10^5^ mL^−1^ for measurements of ^35^S-methionine incorporation. Cells were also collected at mid-day for DNA extraction and PCR amplification of bacterial rDNA.

### 4.3. Determination of Cell Number

Cells were removed aseptically for cell number determination and harvested by centrifugation at 1000× *g*. The recovered cells were resuspended and mixed with a drop of iodine solution (Lugol’s solution). After suitable dilution, using triplicate samples, cells were counted by hemocytometer at 100× magnification, in quadrants 1–4. The average cell count from the four quadrants and the average calculated. Cell number was calculated by multiplying by 10^4^, the dilution factor, and the total volume (mL). Cell numbers were determined in triplicate.

### 4.4. Detection of Bacteria by PCR Amplification

Cells were harvested using centrifugation at 1000× *g* for 10 min, followed by two washes with cold L1. DNA was prepared from 0.8–1.4 × 10^6^ cells using the MP FastSPIN DNA extraction kit (MP Biochemicals, Solon, OH, USA) following the manufacturer’s protocol. DNA was analyzed spectrally using Nanodrop ND-1000 spectrophotometry (Agilent, Santa Clara, CA, USA) and amplified using two universal primers to bacterial 16S ribosomal RNA gene; 27F (5′-AGAGTTTGATCMTGGCTCAG-3′) and Univ1492R (5′-GGTTACCTTGTTACGACTT-3′) [50,51]. Amplification of DNA fragments of the predicted size was confirmed by end-point PCR using 10 ng DNA. Thermal cycling parameters were as follow: initial denaturation at 95 °C for 5 min, followed by 39 cycles of denaturation at 92 °C for 30 s, annealing at 46 °C for 2 min, and extension at 72 °C for 90 s. Extension at 72 °C for 7 min followed the last cycle. Amplicons were fractionated by 1% agarose gel electrophoresis, visualized by ethidium bromide staining and an image generated by ChemiDoc^TM^Touch imaging system (Bio-Rad, Hercules, CA, USA).

### 4.5. ^35^S-Methionine Incorporation

For [^35^S] methionine labeling experiments, cells were used at 4~6 × 10^5^ mL^−1^. The cultures were incubated with [^35^S] methionine (50 μCi·mL^−1^) (PerkinElmer, Boston, MA, USA) for 1 h. After labeling, cells were recovered by centrifugation at 1000× *g* followed by two washes with cold L1 containing 2 mM methionine. The cell pellet was resuspended in 1 mL 10% trichloroacetic acid (TCA) containing 2 mM methionine and kept on ice for 10 min. The samples were incubated at 90 °C for 15 min to deacylate Met-tRNAs. After cooling on ice, the TCA precipitate was filtered through GFC filter paper (Whatman, GE health care Life Science, Little Chalfont, Buckinghamshire, UK), and rinsed with 5% TCA containing 2 mM methionine. Radioactivity was monitored on dried filters by scintillation counting (Beckman, LS6500, Indianapolis, IN, USA). The incorporation of [^35^S] methionine into TCA precipitable radioactivity was corrected for decay of ^35^S and calculated as cpm per 5 × 10^5^ cells.

### 4.6. Effect of Inhibitors of Protein Synthesis on ^35^S-Methionine Incorporation

Cultures (*n* = 3) at 4~6 × 10^5^ mL^−1^ were pre-incubated for 1 h with or without protein synthesis inhibitors: cycloheximide (CHX), 100 µg/mL; chloramphenicol (CAM), 50 µg/mL; emetine (Em), 360 µM; harringtonine (Har), 37.6 µM; sodium arsenite (AsO), 400 µM; thapsigargin (TPG), 1 µM and 5 µM. Cells were incubated with [^35^S] methionine (50 μCi·mL^−1^) for 1 h and processed as described in Section 4.4. Incorporation of [^35^S] methionine into TCA precipitable radioactivity was corrected for ^35^S half-life and expressed per 5 × 10^5^ cells. Data are presented as the percentage of control showing the mean ± S.D (*n* = 3 replicates). Control incubations include an equal volume of the vehicle used for solubilizing. Cycloheximide, chloramphenicol, and sodium arsenite were solubilized in water. Emetine, thapsigargin, and harringtonine were solubilized in dimethyl sulfoxide (DMSO). These were all used at 1/1000 and there was no difference between the water and DMSO.
